# Redox-sensitive polymeric micelles with aggregation-induced emission for bioimaging and delivery of anticancer drugs

**DOI:** 10.1186/s12951-020-00761-9

**Published:** 2021-01-07

**Authors:** Changzhen Sun, Ji Lu, Jun Wang, Ping Hao, Chunhong Li, Lu Qi, Lin Yang, Bin He, Zhirong Zhong, Na Hao

**Affiliations:** 1 Department of Pharmaceutical Sciences, School of Pharmacy, Southwest Medical University, Luzhou, 646000 China; 2 Affiliated Traditional Chinese Medicine Hospital, Southwest Medical University, Luzhou, 646000 China; 3 Biological group, Beijing Huimin School, Beijing, 100032 China; 4 National Engineering Research Center for Biomaterials, Sichuan University, Chengdu, 610064 China

**Keywords:** Polymeric micelles, Redox-sensitive, Aggregation-induced emission, Drug delivery, Bioimaging

## Abstract

**Background:**

Nano-drug delivery systems show considerable promise for effective cancer therapy. Polymeric micelles have attracted extensive attention as practical nanocarriers for target drug delivery and controlled drug delivery system, however, the distribution of micelles and the release of the drug are difficult to trace in cancer cells. Therefore, the construction of a redox-sensitive multifunctional drug delivery system for intelligent release of anticancer drugs and simultaneous diagnostic imaging and therapy remains an attractive research subject.

**Results:**

To construct a smart drug delivery system for simultaneous imaging and cancer chemotherapy, mPEG-ss-Tripp was prepared and self-assembled into redox-sensitive polymeric micelles with a diameter of 105 nm that were easily detected within cells using confocal laser scanning microscopy based on aggregation-induced emission. Doxorubicin-loaded micelles rapidly released the drug intracellularly when GSH reduced the disulfide bond. The drug-loaded micelles inhibited tumor xenografts in mice, while this efficacy was lower without the GSH-responsive disulfide bridge. These results establish an innovative multi-functional polymeric micelle for intracellular imaging and redox-triggered drug deliver to cancer cells.

**Conclusions:**

A novel redox-sensitive drug delivery system with AIE property was constructed for simultaneous cellular imaging and intelligent drug delivery and release. This smart drug delivery system opens up new possibilities for multifunctional drug delivery systems. 
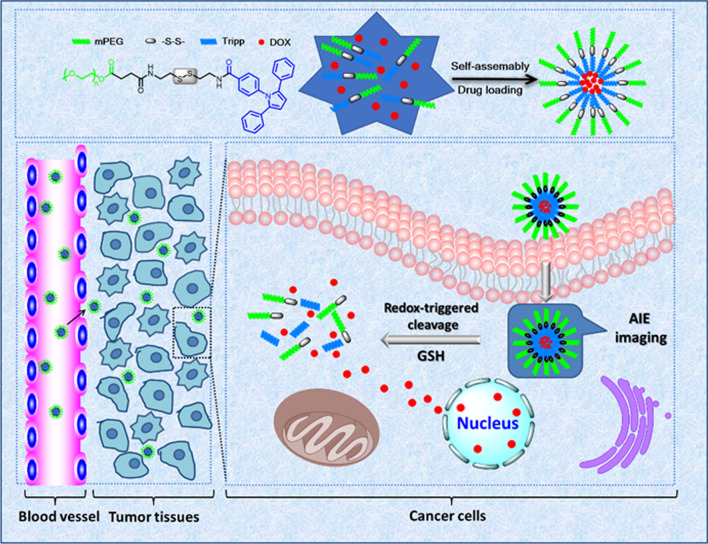

## Background

Nanocarriers have attracted extensive attention as practical methods for target drug delivery and controlled drug delivery system for highly efficient anticancer therapy [1, 2]. Among these carriers, polymeric micelles, self-assembling from amphiphilic block copolymers, are of great interest and importance, because of several merits such as the enhanced water solubility, improved biocompatibility, decreased side effects, passive accumulation of the drugs in the tumor tissues and prolonged circulating time. Although considerable efforts on developing efficient polymeric micelles have been made in recent years, especially, several polymeric micelles have been reached to clinical trials [3, 4], the distribution of micelles and the release of the drug is difficult to trace in cancer cells [5, 6]. Therefore, the development of theranostic nanoparticles with integrated with diagnostic imaging and therapeutic capability is urgent needed for drug delivery systems [7–9]. However, the classical methodology that encapsulates traditional fluorescent dyes into the core of nanoparticles [10–12], often suffered from fluorescence quenching of fluorescence agents in an aggregated state because of the aggregation caused quenching (ACQ) effect, which greatly reduces the fluorescence intensity and impedes the imaging effect, limiting their biomedical applications [13–15]. In 2001, Tang developed a class of novel category of fluorophores with aggregation-induced emission (AIE) characteristics, which exhibit high emission efficiencies in an aggregated state [16, 17]. Recently, the relevant AIE probes have been employed for cell imaging [18–21]. This unique property makes the AIE-active polymeric micelles as the drug nanocarriers available for simultaneous cancer diagnostic and therapy.

When the drug-loaded micelles reaches the target tumor tissues, rapid release of the drug is required to improve antitumor efficacy and circumvent drug resistance in pathological cells. In this regard, smart polymeric micelles with stimuli-sensitive features have been explored for target site-triggered drugs release in response to appropriate environment of tumor tissues [22–26]. Among various stimuli-sensitive antitumor drug nanocarriers, redox-sensitive disulfide linkage contained polymeric micelles have attracted more and more attention for controlled delivery and intelligent release of anticancer drugs. Disulfide linkage of the polymeric micelles can be quickly cleaved by reductive substance glutathione (GSH) via thiol-disulfide exchange [27] at high concentration of GSH in the intracellular environment of tumor cells (approximately 2–10 mM), while relatively stable at a low concentration of GSH in the extracellular environment (approximately 2–20 µM) [24, 28]. Moreover, the GSH concentration in tumor cells is typically at least four times higher than the values of healthy cells [29, 30]. In this context, the construction of a redox-sensitive multifunctional drug delivery system taking advantage of this characteristic for intelligent release of anticancer drugs and simultaneous cancer diagnostic and therapy remains an attractive research subject.

**Scheme 1:**
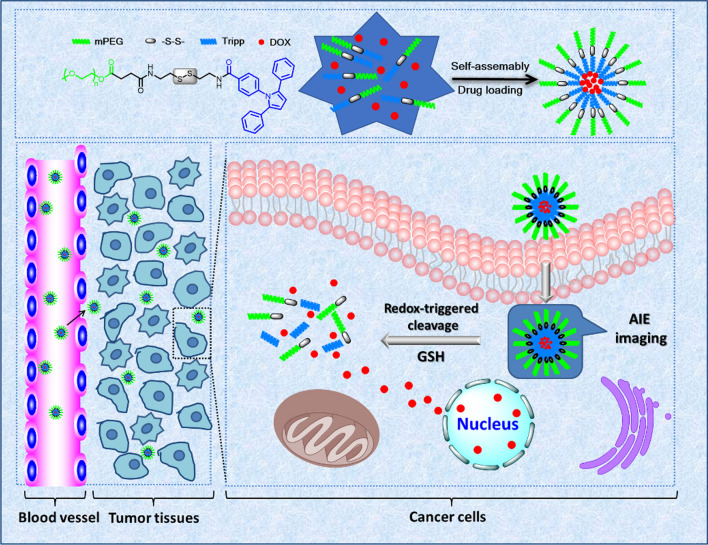
The schematic representation of redox-sensitive copolymers polymeric micelles with AIE imaging for cancer therapy

In this study, a redox-sensitive drug delivery system with AIE property was constructed for simultaneous cellular imaging and intelligent drug delivery and release. We conjugated the AIE fluorophore Tripp to methoxy-PEG via a redox-sensitive disulfide bond. As a control, we also prepared redox-insensitive mPEG-Tripp. We anticipated that the synthetic polymer molecule would self-assemble into micelles in which mPEG would serve as a biocompatible shell that would circulate in the blood for a long time, and the hydrophobic AIE core would allow cellular imaging and loading of hydrophobic anticancer drug such as doxorubicin (DOX). The disulfide bridge, in turn, would help ensure rapid release of the drug within tumor cells but not in the circulation (Scheme 1).

## Materials and methods

### Materials

Methoxypoly (ethylene glycol) (mPEG; Mw 2000 g/mol), succinic anhydride (SAA), chloroform-D (CDCl_3_, 99.8%) and deuterated dimethyl sulphoxide (DMSO-*d*_*6*_) were purchased from Sigma-Aldrich (Shanghai, China); 1-ethyl-(3-dimethylaminopropyl)-carbodiimide hydrochloride (EDC·HCl), methyl 4-aminobenzoate and 1-hydroxy-benzotriazole monohydrate (HOBt), from GL Biochem (Shanghai, China); tetramethylethylenediamine, N,N-diisopropyl ethylamine (DIEA) and 4-dimethylaminopyridine (DMAP), from Aladdin Chemistry (Shanghai, China); 1,3-dicyclohexylcarbodiimide (DCC) and cystamine dihydrochloride, from Asta Tech Biopharm (Chengdu, China); and doxorubicin hydrochloride (DOX·HCl), from Shanghai Yingxuan Chempharm (Shanghai, China). DOX was deprotonated as reported [31]. All solvents were obtained from Chengdu Kelong Chemical (Chengdu, China) and purified before use. Dulbecco's Modified Eagle's Medium (DMEM), Roswell Park Memorial Institute (RPMI) 1640 medium, fetal bovine serum (FBS), and the cell counting kit-8 (CCK-8) were purchased from Life Technologies (Gibco, USA).

### Synthesis of redox-sensitve amphiphiles mPEG-ss-Tripp

#### Synthesis of mPEG-SAA

The mPEG (0.2 g, 0.1 mmol) and SAA (0.05 g, 0.5 mmol) were dissolved in 60 mL of anhydrous CH_2_Cl_2_ with strong stirring. Pyridine (0.4 mL) was added dropwise to the mixture on an ice bath. The mixture was stirred at room temperature for 48 h. An appropriate amount of acetic acid was added to the reaction system under stirring in order to neutralize salts produced in the system. The precipitate was removed by filtration, the filtrate was evaporated and the crude product was precipitated in a large volume of cold diethyl ether. This purification procedure was repeated several times to yield mPEG-SAA.

### Synthesis of mPEG-ss-NH_2_

The mPEG-SAA (0.81 g, 0.4 mmol), EDC·HCl (0.32 g, 1.6 mmol) and HOBT (0.22 g, 1.6 mmol) were dissolved in 60 mL of DMF and stirred at room temperature for 3 h. To the solution was added cystamine dihydrochloride (0.14 g, 0.6 mmol), and the mixture was vigorously stirred at room temperature for 48 h. Then the mixture was filtered and concentrated under reduced pressure. The dark brown residual oil was redissolved in CH_2_Cl_2_, and the organic phase was extracted three times with a saturated aqueous solution of sodium chloride, dried with anhydrous MgSO_4_ overnight and concentrated. The crude product was precipitated three times in cold anhydrous diethyl ether and dried under vacuum to obtain light-yellow solid mPEG-ss-NH_2_.

### Synthesis of mPEG-ss-Tripp

The mPEG-ss-NH_2_ (0.45 g, 0.2 mmol), Tripp-COOH [32] (0.21 g, 0.6 mmol), EDC·HCl (0.16 g, 0.8 mmol) and HOBT (0.11 g, 0.8 mmol) were added to 60 mL of DMF, then DIEA (0.2 mL, 1.2 mmol) was injected into the solution, and the mixture was stirred at room temperature for 48 h. The solvent was evaporated under reduced pressure, and the residue was dissolved in CH_2_Cl_2_. The organic phase was washed three times with a saturated aqueous solution of sodium chloride and dried over anhydrous MgSO_4_. The filtrate was concentrated and precipitated twice in cold diethyl ether to yield mPEG-ss-Tripp.

### Characterization

Products were analyzed in DMSO-*d*_*6*_ using ^1^H NMR (Ascend 400 MHz, Bruker), with tetramethylsilane as an internal reference. Micelle size and size distribution were analyzed by dynamic light scattering (Malvern ZetasizerNano ZS, UK). Micelle morphology was observed using scanning electron microscopy (S4800, Hitachi, Japan). Samples were prepared for electron microscopy by dipping a silicon pellet into a solution of freshly prepared micelles, then drying the pellet overnight at room temperature.

### Preparation of drug-loaded micelles

Drug-loaded micelles DOX/mPEG-ss-Tripp were prepared using a dialysis method. Briefly, freeze-dried amphiphilic mPEG-ss-Tripp (10 mg) and DOX (2.5 mg) were co-dissolved in 1 mL of DMSO, dispersed slowly into 10 mL of deionized water and stirred vigorously overnight. Then, the mixtures were dialyzed against deionized water at 4 °C for 12 h in dialysis tubing (Spectra/Por) with a molecular weight cut-off of 1 kDa. The solution was removed from the dialysis tubing, centrifuged and freeze-dried. Redox-insensitive DOX/mPEG-Tripp micelles were prepared in the same way.

DOX content of drug-loaded micelles was determined by UV/vis spectroscopy (Specord 200 PLUS, Germany) at 480 nm using a standard calibration curve obtained from solutions containing different concentrations of DOX in DMSO. Solutions were shielded from light. Drug loading content (DLC) and encapsulation efficiency (DLE) were calculated according to the following formulas:

$$ {\text{DLC }}\left( \% \right) \, = \, \left( {{\text{weight of loaded DOX}}/{\text{weight of drug}} - {\text{loaded micelle}}} \right) \, \times { 1}00\% $$

$$ {\text{DLE }}\left( \% \right) \, = \, \left( {\text{weight of loaded DOX/weight of drug in feeding}} \right) \, \times { 1}00\% $$

### In vitro drug release

Profiles of DOX release from micelles were investigated in phosphate-buffered saline (PBS; pH 7.4 and pH 5.5, ionic strength = 0.01 M) alone or supplemented with 10 mM GSH. Briefly, 1 mL of drug-loaded micelles were transferred into dialysis tubes (Spectra/Por) with a molecular weight cut-off of 1 kDa and immersed in 25 mL of release medium in vials with continuous shaking at 120 rpm and 37 °C. At predetermined intervals, 1 mL of release medium was taken out and replaced with an equal volume of fresh medium. The released DOX was detected using a fluorescence spectrometer (F-7000, Hitachi, Japan) with excitation at 480 nm and emission at 550 nm. The release experiments were conducted in triplicate under sink conditions, and average values with standard deviations were presented.

### In vitro cytotoxicity and antitumor efficacy

The biocompatibility of blank micelles was evaluated using the CCK-8 assay. 293 T human embryonic kidney cells and 4T1 breast cancer cells were separately seeded in 96-well plates (6 × 10^3^ cells per well) in 100 μL of culture medium and incubated for 24 h.

Medium was removed and replaced with 100 µL of medium containing different concentrations of blank micelles or DOX-loaded micelles. Then the cells were incubated for another 48 h, the medium was removed, the wells were rinsed three times with PBS, and CCK-8 solution (100 µL, volume fraction, 10%) was added to each well. The cells were incubated another 2 h in the dark, and absorbance was measured at 450 nm using a microplate reader (Thermo Scientific MK3, USA).

Antitumor activity of drug-loaded micelles against 4T1 cells was also evaluated using the CCK-8 assay. Cells were seeded and incubated for 24 h in 96-well plates as described above, then free DOX·HCl or DOX-loaded micelles in culture medium were added at various DOX concentrations (0.001–100 μg/mL) and the cells were incubated another 48 h. After that, the cells were subjected to the CCK-8 assay as described above.

### Cellular uptake of micelles

Uptake of micelles by 4T1 cells was analyzed using confocal laser scanning microscopy and flow cytometry. Cells in logarithmic growth were seeded in 35-mm confocal dishes (2 × 10^4^ mL^−1^ cells per well) and incubated for 24 h. Then the medium was replaced with blank or DOX-loaded micelles (10 μg/mL DOX) in culture medium and cells were incubated for 1 or 3 h. The medium was removed and cells were washed three times with cold PBS, then examined using confocal laser scanning microscopy with excitation at 480 nm and emission at 590 nm.

For the flow cytometry tests, 4T1 cells were seeded in 6-well plates (1 × 10^5^ mL^−1^ cells per well), incubated for 24 h, then treated for 1 or 3 h with free DOX·HCl or DOX-loaded micelles (10 μg/mL DOX). After that, medium was removed and cells were washed three times in PBS and harvested by trypsin treatment. Finally, cells were centrifuged (1000 rpm, 5 min), resuspended in PBS, and analyzed using flow cytometry (BD FACSCanto II, USA).

### In vivo toxicity and antitumor efficacy

All animal experiments were performed according to institutional guidelines and the recommendations of the US National Institutes of Health for the care and use of research animals. Male BALB/c mice (18–20 g) were purchased from West China Experimental Animal Culture Center of Sichuan University (Chengdu, China). 4T1 cells (1 × 10^6^) were injected subcutaneously into the right flank, and when the inoculated tumors reached a volume of 100–200 mm^3^, the animals were randomized to receive one of the following treatments (n = 8 per group): saline, DOX·HCl, DOX/mPEG-ss-Tripp micelles, or DOX/mPEG-Tripp micelles (DOX always at 5 mg/kg body). Treatments were delivered intravenously via the tail vein every 3 days for 4 treatments. Tumor volume and body weight were monitored at prescribed time intervals, and tumor volume was calculated using the formula: V (mm^3^) = 1/2 × ab^2^, where a and b are the largest and smallest diameters of the tumor, respectively.

At 21 d after the fourth treatment, all mice were sacrificed. Organs including heart, liver, spleen, lung, kidney and tumor were excised from each mouse and fixed in 4% formaldehyde. Representative tissues were paraffin-embedded, sectioned, stained with hematoxylin and eosin and examined under a light microscope. Blood was assayed using standard serum tests performed by Chengdu Lilai Biotechnology (Chengdu, China) Tumor sections were immunostained with monoclonal antibody against Ki-67 to assess tumor proliferation, while sections were assayed by terminal deoxynucleotidyl transferase-mediated deoxyuridine triphosphate nick-end labeling (TUNEL) to assess the level of apoptosis.

### Statistical analysis

All data are presented as mean ± standard deviation (SD). Inter-group differences were assessed for significance using Student’s t test, and p < 0.05 was defined as significant.

## Results and discussion

### Characterization of mPEG-ss-Tripp and mPEG-Tripp

**Scheme 2:**
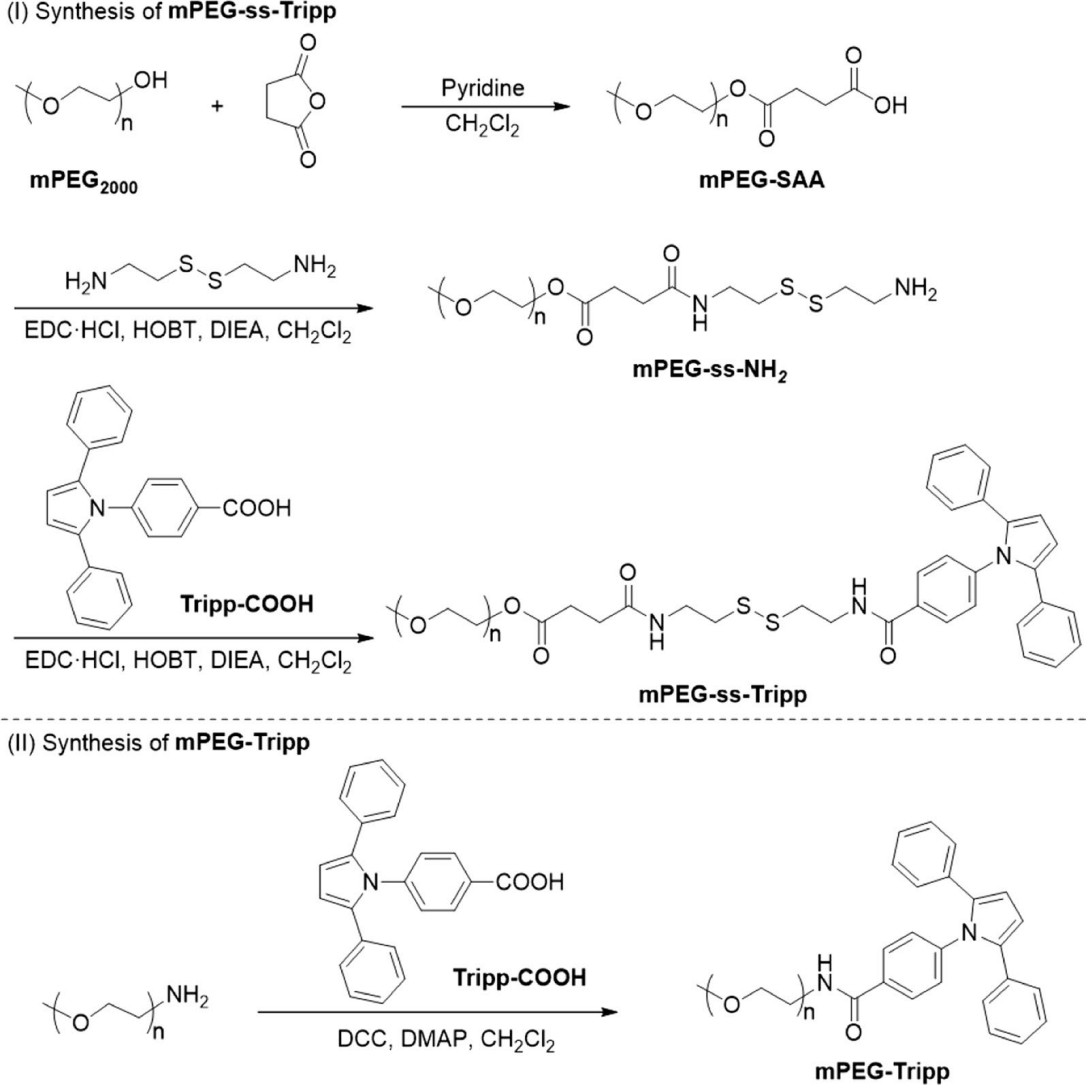
Synthesis of the mPEG-ss-Tripp (I) and mPEG-Tripp (II) copolymer

**Fig. 1.**
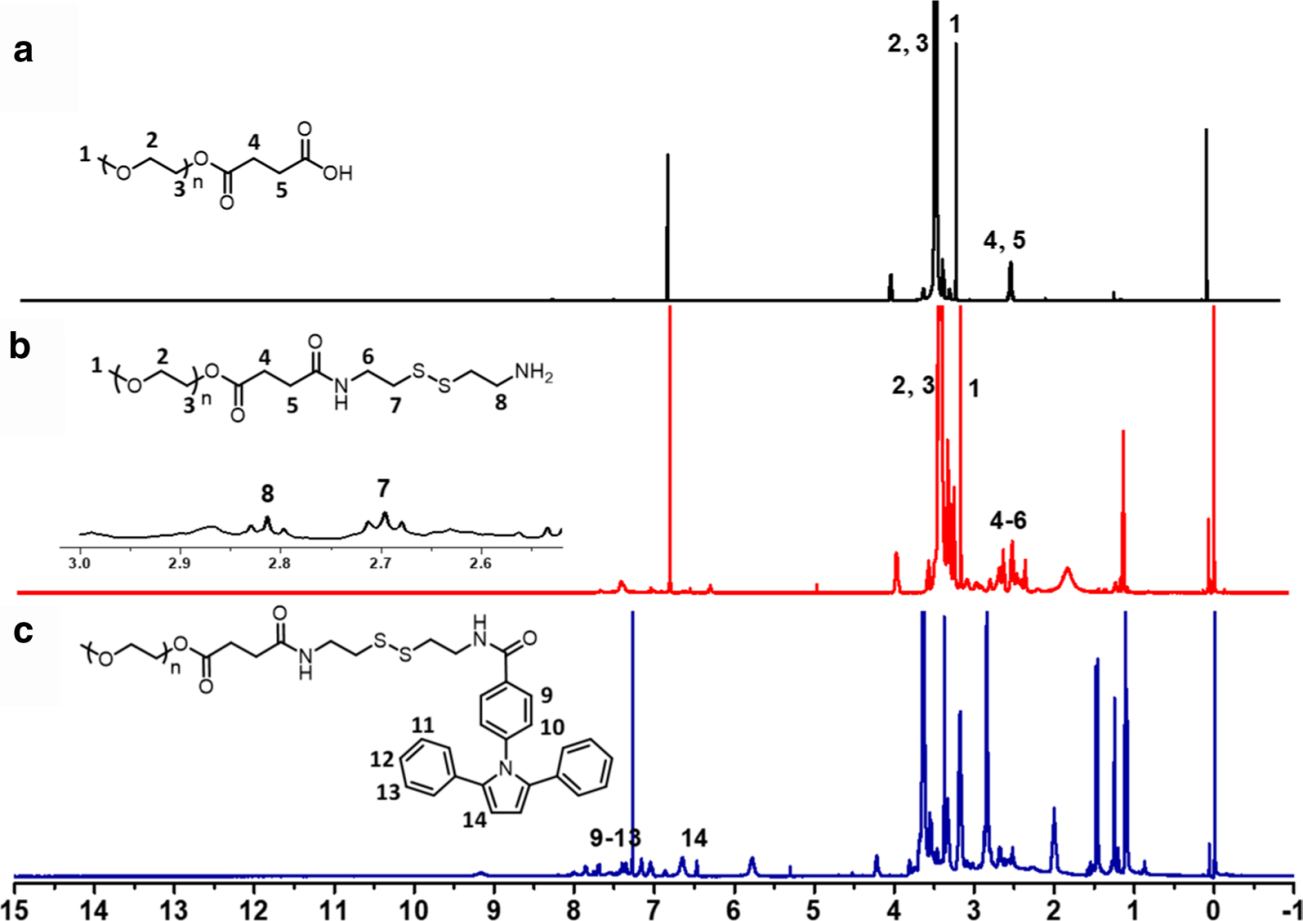
^1^H NMR spectra of mPEG-SAA (CDCl_3_) (**a**); mPEG-ss-NH_2_ (DMSO-*d*_*6*_) (**b**) and mPEG-ss-Tripp (DMSO-*d*_*6*_) (**c**)

The conjugates mPEG-ss-Tripp and mPEG-Tripp were synthesized conveniently (Scheme 2), and the chemical structures of intermediates and final copolymers were confirmed by ^1^H NMR. The protons of mPEG-SAA showed chemical shifts at δ = 3.35–3.40 ppm (1), δ = 3.50–3.80 ppm (2, 3) and δ = 2.55–2.67 ppm (4, 5) (Fig. 1a). Conjugation with cystamine via an amido linkage gave peaks at δ= 2.60–3.00 ppm (7, 8), which were assigned to the protons of –CH_2_CH_2_ in cystamine (Fig. 1b). The appearance of peaks at δ = 7.70–7.80 ppm, attributed to an amide bond, confirmed that the cystamine segment was introduced into mPEG-SAA. The appearance of peaks at δ = 6.50 ppm (14) and δ = 7.20–7.89 ppm (9–13), attributed to the pyrrole and benzene rings, respectively (Fig. 1c), confirmed the synthesis of the amphiphilic polymer mPEG-ss-Tripp. In mPEG-Tripp, peaks at δ = 3.35 ppm (1) and δ = 3.6 ppm (2) were assigned, respectively, to protons in the terminal CH_3_ and OCH_2_CH_2_ in PEG. The other two peaks at δ = 6.50 ppm (6) and δ = 7.08–7.73 ppm (3, 4, 5, 7, 8) were attributed, respectively, to the pyrrole and benzene rings (Additional file 1: Figure S1).

### Preparation of blank and DOX-loaded micelles

**Fig. 2:**
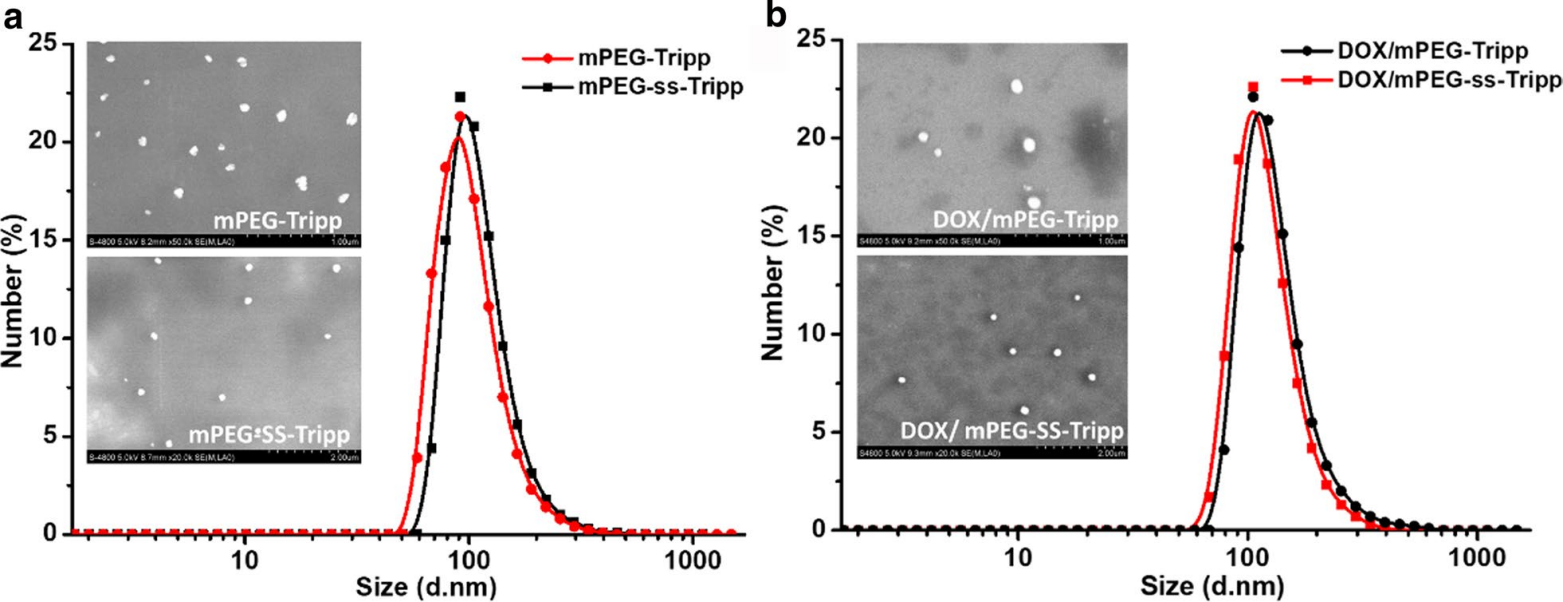
DLS and SEM images of blank micelles (**a**); DOX-loaded micelles (**b**)

Based on dynamic light scattering, hydrodynamic diameters of blank micelles were 102 nm for mPEG-ss-Tripp and 99 nm for mPEG-Tripp (Fig. 2a), and loading the two micelles with DOX increased their respective diameters to 122 and 117 nm (Fig. 2b). With or without drug, micelles showed narrow size distributions, with a polydispersity index of 0.17–0.21. Scanning electron microscopy showed uniform, spherical sizes that were consistent with the results of dynamic light scattering. These sizes can help nanoparticles to evade scavenging by mononuclear phagocytes as well as to target tumors passively through enhanced permeability and retention [33–35].

Micelles of mPEG-ss-Tripp and mPEG-Tripp showed respective DLCs of 10.5% and 10.1%, and respective DLEs of 71.6% and 69.5%. The relatively high DLC of micelles is due to the introduction of π-conjugated moieties (Tripp), which enhances π-π stacking between DOX and amphiphilic polymers. After excitation at 485 nm, free DOX·HCl emitted intense fluorescence from 500 to 700 nm, while DOX/mPEG-ss-Tripp and DOX/mPEG-Tripp at the same DOX concentration (25 μg/mL) emitted much weaker signal (Additional file 1: Figure S2). This quenching is presumably due to the π-π stacked DOX [36].

### AIE behavior of mPEG-ss-Tripp

**Fig. 3:**
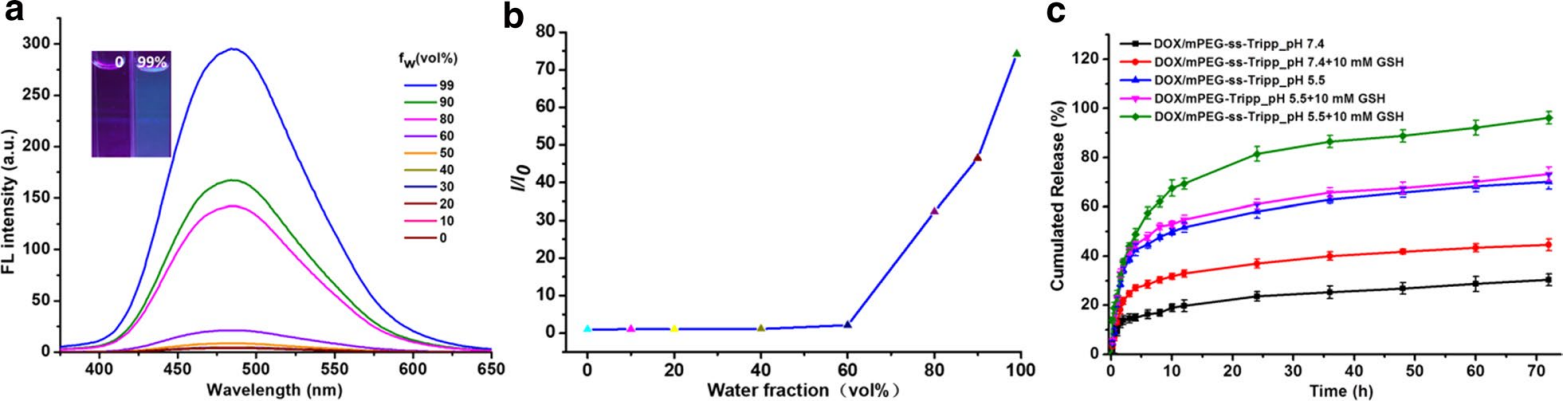
The fluorescent spectra of mPEG-ss-Tripp in DMSO/water mixtures with different fractions of water (f_w_), insert image showing fluorescence of mPEG-ss-Tripp solutions dissolved in pure DMSO and 99% water (**a**); plot of fluorescent intensity of mPEG-ss-Tripp versus water content of DMSO/water mixture, I = fluorescent intensity of mPEG-ss-Tripp in mixed solution, I_0_ = fluorescent intensity of mPEG-ss-Tripp in pure DMSO (λex = 330 nm) (**b**); the release profiles of DOX-loaded micelles triggered with or without GSH at pH 7.4 and pH 5.5, means ± SD (n = 3) (**c**)

Since the Tripp-COOH groups, as the hydrophobic moieties in the amphiphile mPEG-ss-Tripp, are trapped inside the micellar cores during micelle formation, the aggregation state of Tripp-COOH molecules should trigger AIE. We confirmed this in vitro using a fluorescence assay. The mPEG-ss-Tripp dissolved in DMSO showed weak fluorescence, which increased dramatically when water was added to the DMSO solution (Fig. 3a). When the water fraction reached 99% (v/v), fluorescence intensity of mPEG-ss-Tripp was nearly 74-fold stronger than in 100% DMSO (Fig. 3b). These results confirm the AIE of mPEG-ss-Tripp micelles.

### In vitro drug release

Antitumor drug was expected to rapidly release as soon as drug-loaded micelles reached tumor tissue. The GSH triggered drug release of DOX-loaded micelles was investigated in PBS buffer solution at pH 7.4 and pH 5.5 with or without GSH (Fig. 3c). The amount of DOX released from mPEG-ss-Tripp micelles in the medium at pH 7.4 contained 10 mM GSH was less than 45% after 72 h, while more than 70% was released in acidic medium at pH 5.5 in the absence of GSH. Remarkably, drug release was quicker in acidic medium in the presence of 10 mM GSH, with more than 96% of drug release after 72 h. These results suggest that, as desired, our nanosystem delivers drug cargo selectively within cells, in response to the microenvironment of tumor and enhanced antitumor efficacy.

### Change in micelle size in response to GSH

**Fig. 4:**
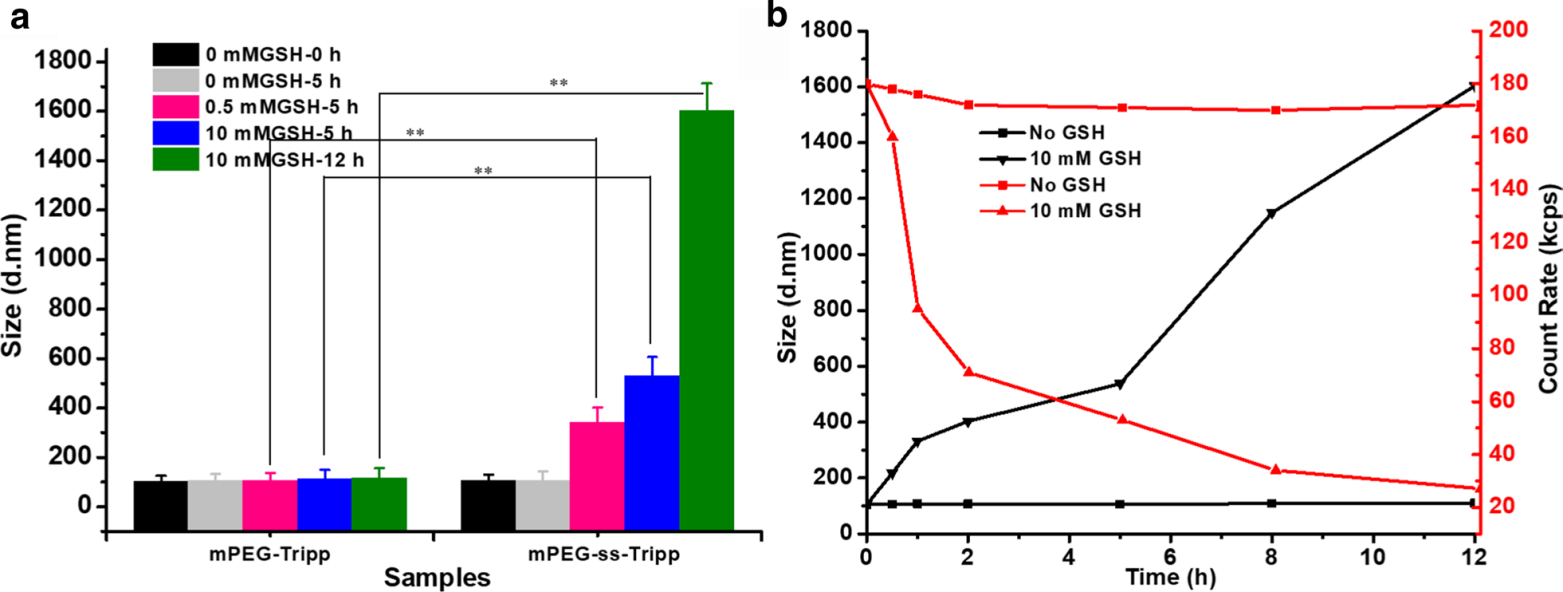
The size changes of mPEG-Tripp and mPEG-ss-Tripp micells in different concentration of GSH solution (**a**); the size changes and the count rate of mPEG-ss-Tripp micells over time with or without 10 mM GSH (**b**), ** P < 0.01

Dynamic light scattering showed that mPEG-ss-Tripp micelles swelled rapidly in the presence of GSH: in the presence of 0.5 mM GSH, average size increased from 105 to 342 nm within 5 h; in the presence of 10 mM GSH, average size increased from 342 to 531 nm in 5 h, and to > 1600 nm in 12 h (Fig. 4a). In contrast, the size of mPEG-Tripp micelles changed little even after 12 h in the presence of 10 mM GSH. Consistent with GSH-triggered micelle opening and drug release, the count rate of the mPEG-ss-Tripp micelles decreased with time and with increasing GSH concentration (Fig. 4b). These results suggest that 10 mM GSH can trigger complete dissolution of mPEG-ss-Tripp micelles.

### In vitro cytotoxicity of micelles

**Fig. 5:**
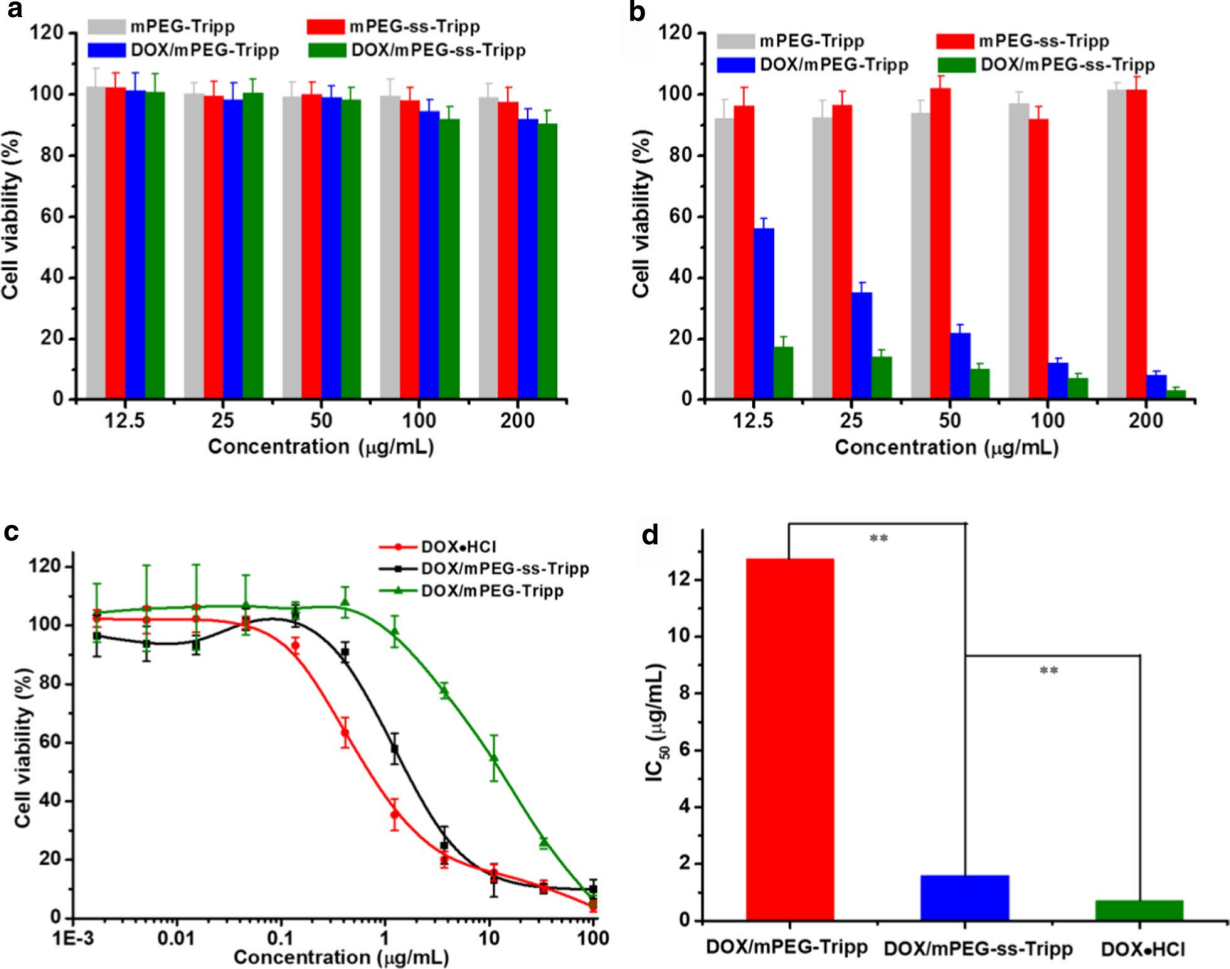
In vitro cell viability and antitumor efficiency. Cell viability versus concentrations of various blank micelles and DOX-loaded micelles against the normal cell line 293 T (**a**) and the tumor cell line 4T1 (**b**); Cell viability of 4T1 cells versus concentrations of various formulations with incubation for 48 h (means ± SD, n = 3) (**c**); IC_50_ values of various formulations to 4T1 cells (**d**), ** P < 0.01

293 T and 4T1 cells were utilized to evaluate the potential cytotoxicity of blank micelle and DOX-loaded micelles based on the CCK-8 assay (Fig. 5a and b). Both cells showed great cell viability after 48 h incubation even the concentration of blank micelles increased to 200 µg/mL. In addition, DOX-loaded micelles also exhibited negligible toxicity against 293 T cell. However, great cell toxicity of 4T1 cells treated with DOX-loaded micelles was observed, and DOX/mPEG-Tripp micelles showed lower inhibition efficiency than that of DOX/mPEG-ss-Tripp micelles. Therefore, the difference in potency between cancer cell lines and normal cell lines indicated good tumor selectivity of DOX/mPEG-ss-Tripp micelles. At the same time, DOX-loaded micelles reduced the viability of 4T1 cells, although their half-maximal inhibitory concentrations (IC_50_) were higher than for free DOX (mPEG-ss-Tripp, 1.65 μg/mL; mPEG-Tripp, 12.74 μg/mL; free DOX·HCl, 0.71 μg/mL (Fig. 5c and d). The greater anticancer activity of free DOX likely reflects its ability to enter cells by passive diffusion. The much greater anticancer activity of mPEG-ss-Tripp than mPEG-Tripp likely reflects that cleavage of the disulfide bond within cells triggers faster drug release from the micelle interior than simple diffusion.

### Intracellular imaging of AIE micelles in vitro

**Fig. 6:**
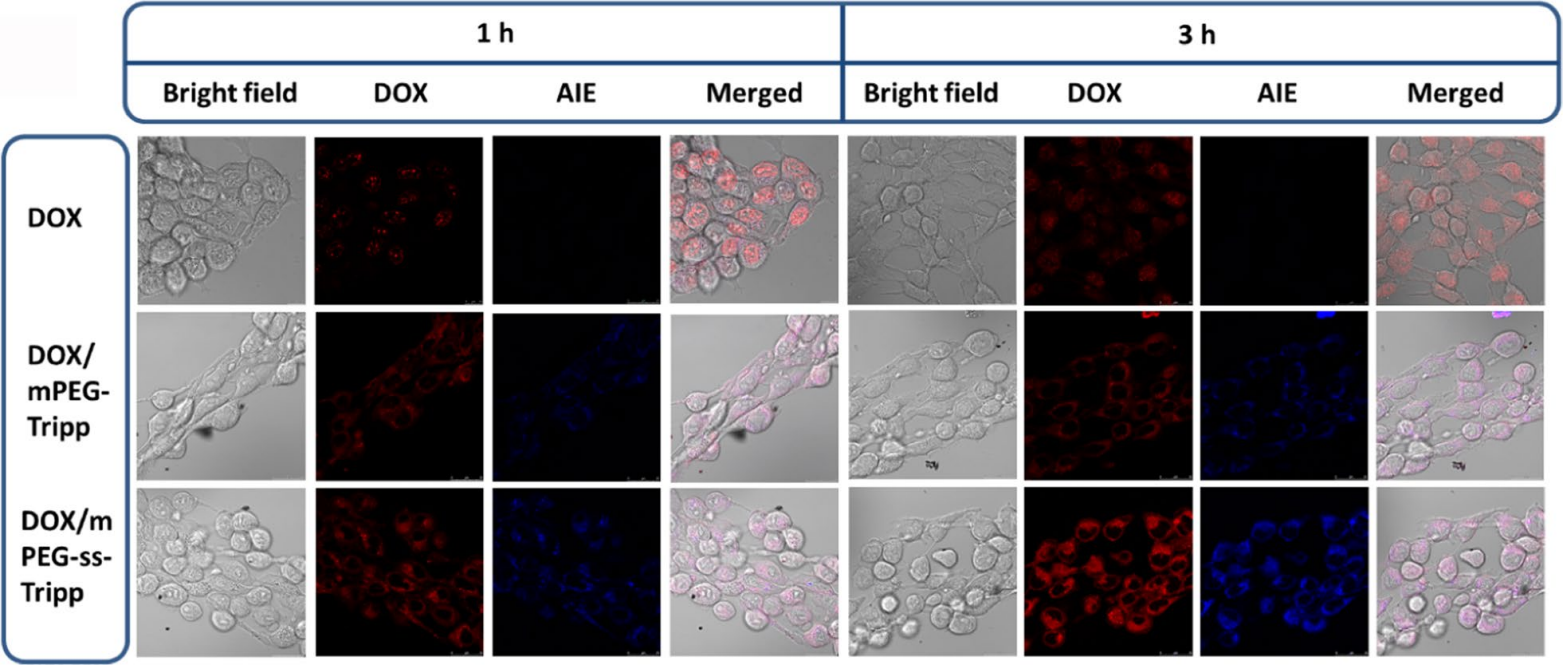
The confocal laser scanning microscopy images of 4T1 cells incubated with DOX∙HCl, DOX/mPEG-Tripp micells, and DOX/mPEG-ss-Tripp micelles for 1 h and 3 h, the scale bar was 20 μm

Uptake of micelles and intracellular release of drug were analyzed using confocal fluorescence microscopy and flow cytometry on the basis of AIE signal. Within 1 h of incubation with blank or DOX-loaded micelles, blue AIE fluorescence was clearly visible within the cytoplasm of 4T1 cells (Fig. 6a), suggesting rapid internalization of micelles via endocytosis. Cells incubated with free DOX·HCl showed red fluorescence mainly in nuclei, reflecting the excellent aqueous solubility of DOX and its ability to enter cells and nuclei by passive diffusion. However, for the DOX-loaded micelles, most red fluorescence of DOX was concentrated in cytoplasm; only weak red fluorescence was observed in nuclei. Both red and blue fluorescence intensity were higher at 3 h than at 1 h, confirming that more drug-loaded micelles were internalized into cells and sustainably released the drug intracellularly. These experiments confirm that DOX-loaded micelles can be used for bioimaging and drug delivery.

**Fig. 7:**
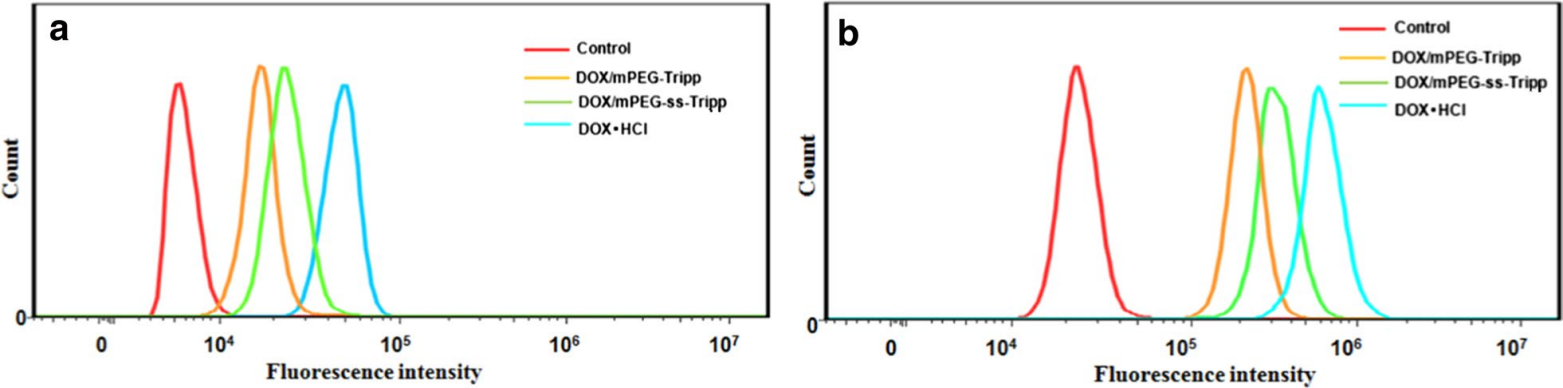
The flow cytometry histogram profiles of 4T1 cells incubated with DOX∙HCl, DOX/mPEG-Tripp micells and DOX/mPEG-ss-Tripp micelles for 1 h (**a**) and 3 h (**b**), the DOX concentration was 10 μg/mL

The results of confocal microscopy were confirmed using flow cytometry. While control cells showed only autofluorescence, cells treated with DOX·HCl showed the strongest fluorescence, followed by cells treated with mPEG-ss-Tripp and finally cells treated with mPEG-Tripp (Fig. 7a and b).

### In vivo toxicity and antitumor efficacy of micelles

**Fig. 8:**
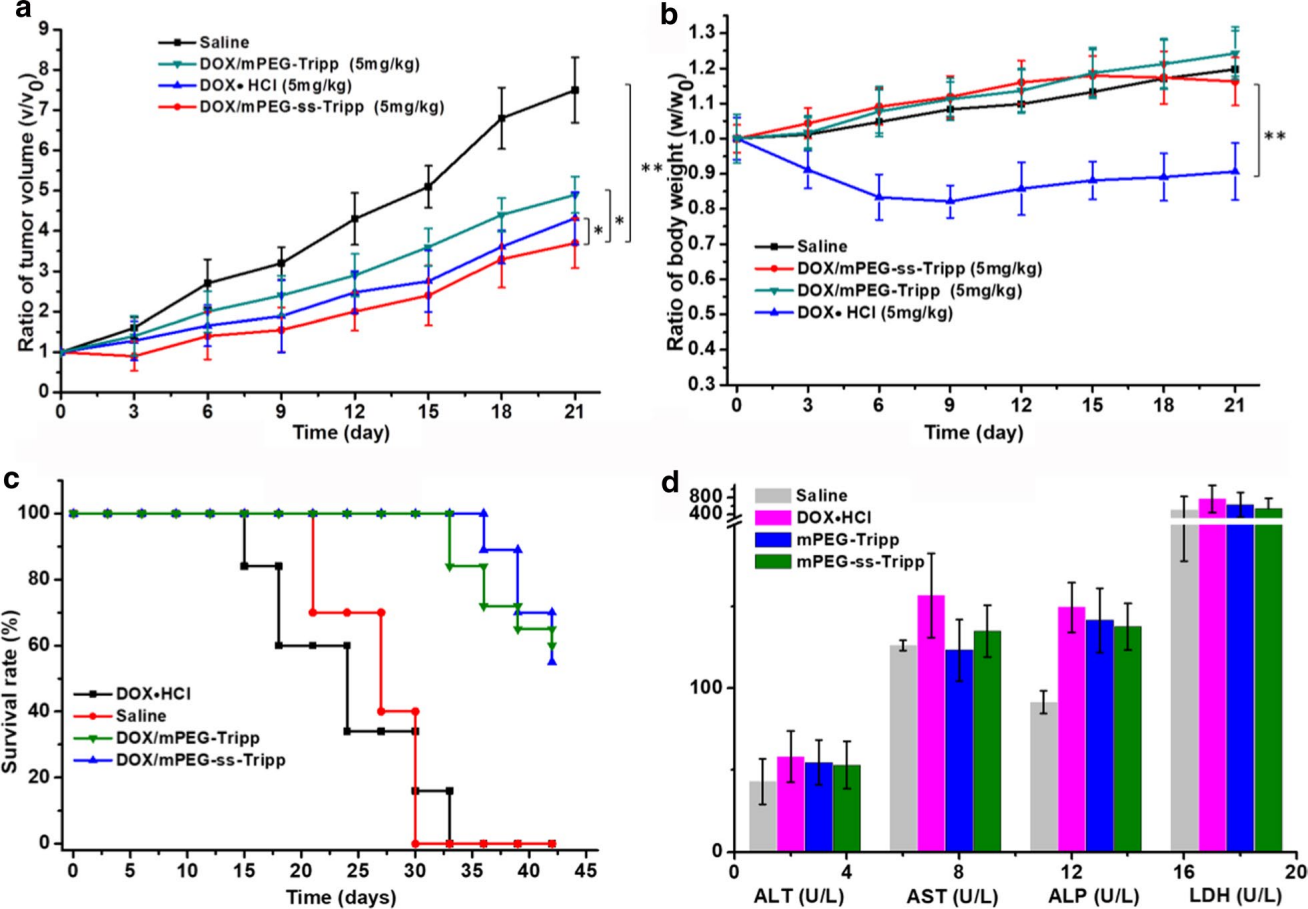
The in vivo anticancer activity of DOX-loaded micelles. The volumes of tumors treated with DOX-loaded micelles (**a**); body weights of the mice treated with DOX-loaded micelles (**b**); survival rate of tumor-bearing mice (**c**); histogram of serum biochemical test of DOX- loaded micelles (**d**). Data was presented as means ± SD (n = 8), * P < 0.05, ** P < 0.01

Tumor-bearing BALB/c mice were treated every 3 days with saline, DOX·HCl or DOX-loaded micelles. By 21 days of treatment, the tumor volume in the saline group had increased rapidly (Fig. 8a), indicating that the saline did not have any therapeutic effect. DOX·HCl and DOX-loaded micelles, in contrast, strongly inhibited tumor growth. Notably, treatment with mPEG-ss-Tripp group showed better anticancer activity than mPEG -Tripp group. In addition, the picture of harvested tumors and the average tumor weight of each group further confirmed the greater antitumor efficacy of mPEG-ss-Tripp micelles (Additional file 1: Figure S3A and B). Over the same period, the DOX induced toxicity was reflected by body weight change of mice (Fig. 8b). There was no body weight loss for saline treated mice. However, a severe body weight loss could be observed for the free DOX treated mice. On the contrary, the body weight changes of mice did not show distinct difference in DOX-loaded micelles treatment group and the control group, indicating a significant lower toxicity than free DOX. Consistent with this idea, all mice treated with DOX-loaded micelles survived for the entire period of 33 days, whereas 40% of mice treated with free DOX·HCl died within 18 days (Fig. 8c). Therefore, it could be confirmed that DOX-loaded micelles would be a good candidate for tumor therapy with an improved antitumor efficacy and decreased side effects.

Serum biochemistry assays confirmed that free DOX·HCl was strongly toxic, which was reflected in elevated aspartate transaminase (AST), alanine aminotransferase (ALT) and alkaline phosphatase (ALP) (Fig. 8d). Indices in animals treated with DOX-loaded micelles were close to those in the saline group, indicating that the DOX-loaded micelles did not obviously harm tumor-bearing mice.

**Fig. 9:**
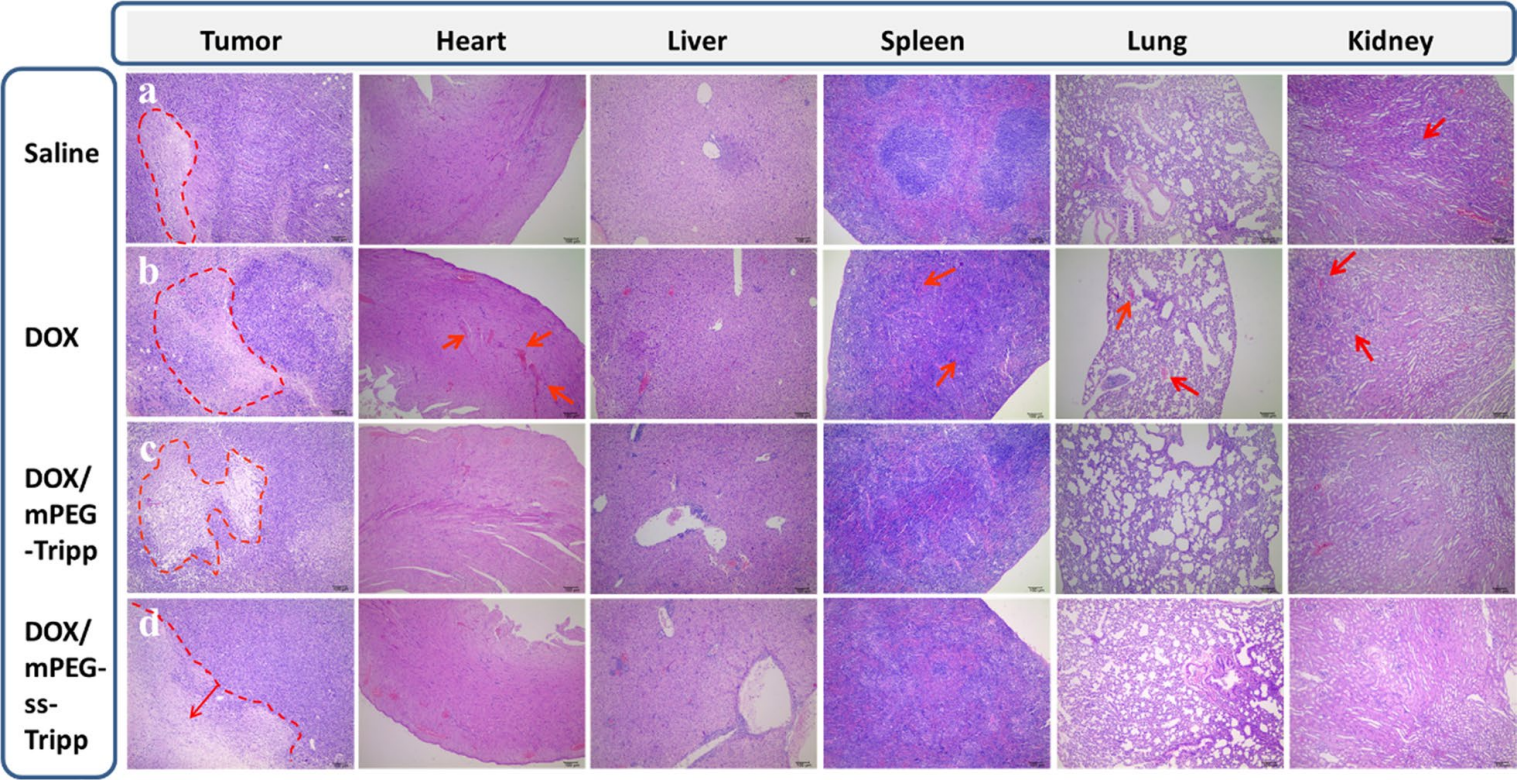
Histological analysis of the main organs of tumor bearing mice treated with different methods by H&E staining (× 400). Each group of BALB/c mice (male, n = 8) was intravenously administered four times at a three-day interval at a dose of 5 mg/kg (DOX)

Hematoxylin–eosin staining of tumor tissue showed nearly no apoptosis or necrosis in the saline group, while it showed tumor necrosis with hemorrhage in the groups treated with free DOX·HCl or DOX-loaded micelles (Fig. 9). Staining of slices indicated that obvious cardiotoxicity could be observed in mice treated with free DOX, whereas no obvious abnormity was observed for the cardiac tissue treated with DOX-loaded micelles. Meanwhile, compared with DOX-loaded micelles, free DOX exhibited more focal necrosis and inflammation in lungs, spleen, and kidney, such as lobular pneumonia in lungs, white pulp atrophy and splenic corpuscle disappearance in spleen, swelling and shrinkage or disappearance of kidney glomerular cells.

### Immunohistochemistry and TUNEL staining

**Fig. 10:**
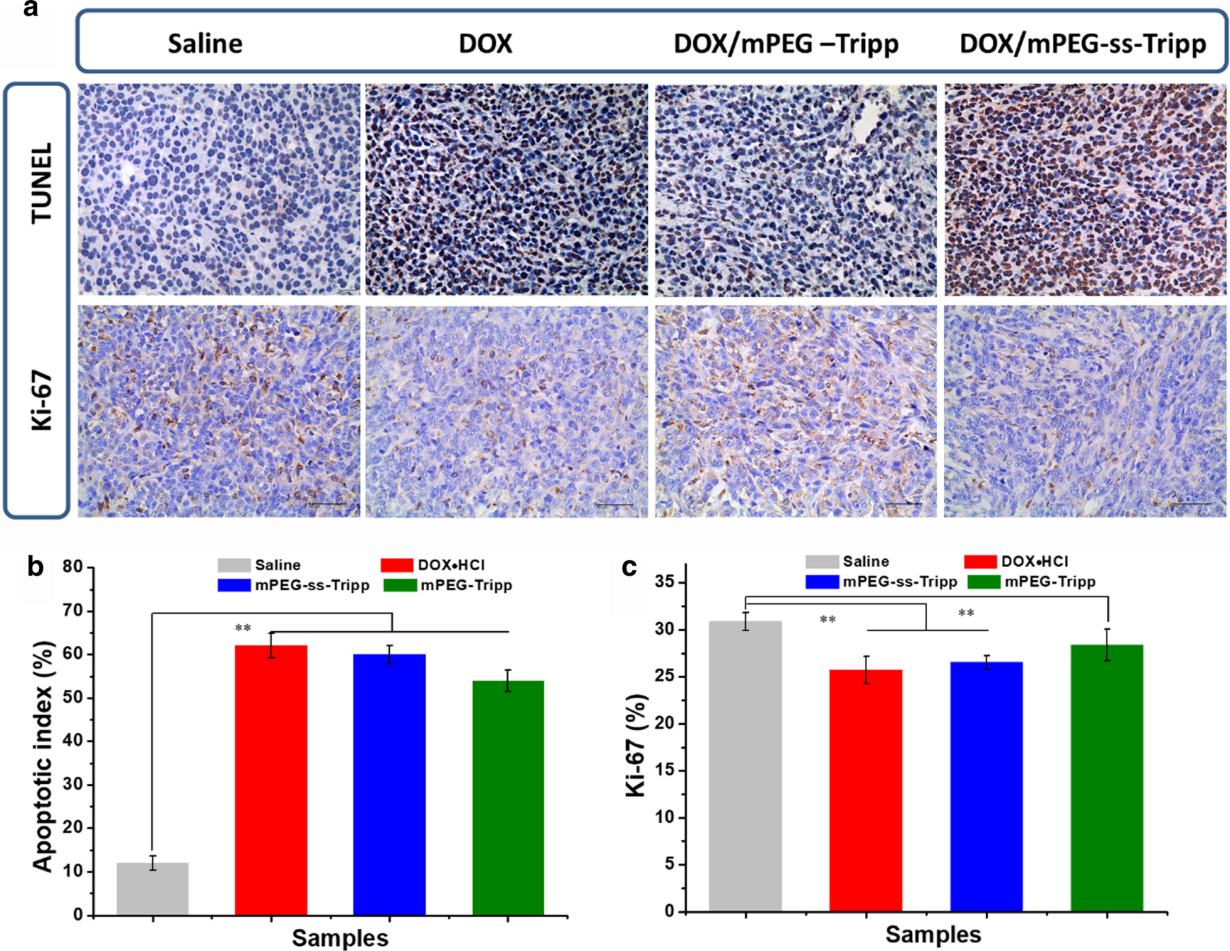
The TUNEL immunohistochemical (IHC) staining and the Ki-67 antigen staining of tumor tissues (× 400). Data was presented as mean ± SD (n = 6), ** P < 0.01. The dose of DOX was 5 mg/kg

Tissue sections stained against Ki-67 antigen as an index of tumor proliferation showed that mPEG-ss-Tripp micelles suppressed proliferation more than other treatments (Fig. 10). These results confirm that the redox-sensitive of the drug delivery system can improve therapeutic efficacy in vivo.

These in vivo experiments indicate that mPEG-ss-Tripp micelles lead to substantially lower toxicity than free DOX·HCl and greater efficacy than redox-insensitive mPEG-Tripp micelles.

## Conclusions

We have designed a novel redox-sensitive amphiphilic polymer with AIE character to realize a drug delivery system for simultaneous cellular imaging and cancer therapy. When loaded with DOX, the amphiphilic polymer can self-assemble into drug-loaded micelles, which release their therapeutic cargo rapidly in response to reduction of the disulfide linkage at the high GSH concentrations inside cells. The micelles can be tracked within cells by virtue of their AIE. Removing the redox-sensitive disulfide bridge makes drug delivery slower and less effective against tumors. These results open up new possibilities for multifunctional drug delivery systems.

## Supplementary Information

**Additional file 1**: **Figure S1.** ^1^H NMR spectra of mPEG-Tripp. **Figure S2.** Fluorescence spectra of micelles in aqueous solution at 485 nm excitation wavelength. **Figure S3.** Image of tumors after treatment for three weeks with different formulations after 21 days.

## Data Availability

All data generated or analyzed during this study are included in this published article and its Additional file 1.

## Content for publication

All authors agreed to submit this manuscript.
