# Supplementary material for: Redox-sensitive polymeric micelles with aggregation-induced emission for bioimaging and delivery of anticancer drugs
Source: J Nanobiotechnology. 2021 Jan 7;19:14. doi: 10.1186/s12951-020-00761-9 (PMC7791786; doi:10.1186/s12951-020-00761-9)
Supplement: Supplementary file 1 — Additional file 1: Figure S1. 1H NMR spectra of mPEG-Tripp. Figure S2. Fluorescence spectra of micelles in aqueous solution at 485 nm excitation wavelength. Figure S3. Image of tumors after treatment for three weeks with different formulations after 21 days. [file 12951_2020_761_MOESM1_ESM.docx]

Supporting Information

Redox-Responsive Polymeric Micelles with Aggregation-Induced Emission for Bioimaging and Delivery of Anticancer Drug

Changzhen Sun^,†a,b^ Ji Lu,^†a^ Jun Wang,^a^ Ping Hao,^c^ Chunhong Li,^a^ Lu Qi,^a^ Lin Yang,^a^ Bin He,^d^ Zhirong Zhong^*a^ and Na Hao^*a^

*^a^Department of Pharmaceutical Sciences, School of Pharmacy, Southwest Medical University, Luzhou 646000, China.*

*^b^Affiliated Traditional Chinese Medicine Hospital, Southwest Medical University, Luzhou 646000, China.*

*^c^Biological group, Beijing Huimin School, Beijing 100032, China.*

*^d^National Engineering Research Center for Biomaterials, Sichuan University, Chengdu 610064, China.*

**Synthesis of mPEG-Tripp**

The mPEG (0.2 g, 0.1 mmol), DMAP (0.01 g, 0.1 mmol) and Tripp-COOH (0.34 g, 1.0 mmol) were dissolved in 30 mL of anhydrous CH2Cl2 with vigorous stirring under nitrogen. A solution of DCC (0.412 g, 2.0 mmol) in CH2Cl2 (10 mL) was added dropwise to the mixture on an ice bath. The mixture was stirred at room temperature for 48 h. The white solid dicyclohexylurea (DCU) precipitate was removed by filtration, and the filtrate was concentrated and precipitated with a large volume of cold diethyl ether. This purification procedure was repeated several times, and the product was vacuum-dried at 40 °C.

Figure S1. ^1^H NMR spectra of mPEG-Tripp (DMSO-*d*_6_).

Figure S2. Fluorescence spectra of micelles in aqueous solution at 485 nm excitation wavelength.


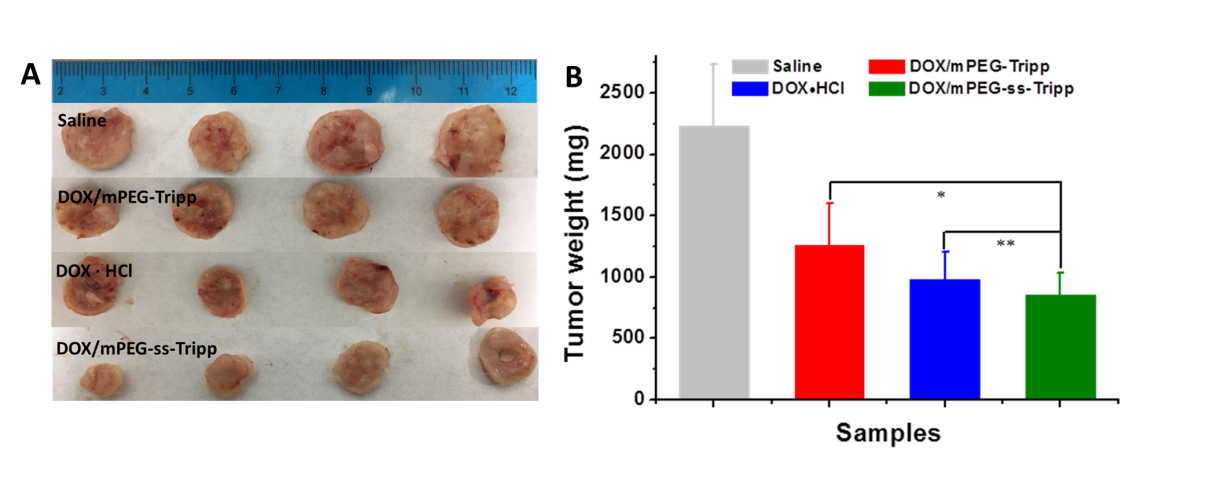


Figure S3. Image of tumors of mice treated with different formulations after 21 days (A); The tumor weight of mice treated with saline, DOX•HCl, and DOX-loaded micelles after 21 days (B) (n = 8, * P < 0.05, ** P < 0.01).
